# Sex differences in fetal Doppler parameters during gestation

**DOI:** 10.1186/s13293-021-00370-7

**Published:** 2021-03-10

**Authors:** Dakshita Jagota, Hannah George, Melissa Walker, Anjana Ravi Chandran, Natasha Milligan, Shiri Shinar, Clare L. Whitehead, Sebastian R. Hobson, Lena Serghides, W. Tony Parks, Ahmet A. Baschat, Christopher K. Macgowan, John G. Sled, John C. Kingdom, Lindsay S. Cahill

**Affiliations:** 1 Department of Chemistry, Memorial University of Newfoundland, 283 Prince Philip Drive, St. John’s, NL A1B 3X7 Canada; 2 Mount Sinai Hospital, Toronto, Ontario Canada; 3 Department of Obstetrics and Gynecology, University of Toronto, Toronto, Ontario Canada; 4 Division of Cardiology, Department of Paediatrics, The Hospital for Sick Children, Toronto, Ontario Canada; 5 Pregnancy Research Centre, Department of Obstetrics and Gynaecology, Royal Women’s Hospital, Parkville, Australia; 6 Toronto General Hospital Research Institute, University Health Network, Toronto, Ontario Canada; 7 Department of Immunology and Institute of Medical Sciences, University of Toronto, Toronto, Ontario Canada; 8 Women’s College Research Institute, Women’s College Hospital, Toronto, Ontario Canada; 9 Department of Pathology, Mount Sinai Hospital, Toronto, Ontario Canada; 10 Department of Laboratory Medicine and Pathobiology, University of Toronto, Toronto, Ontario Canada; 11 Centre for Fetal Therapy, Johns Hopkins Medicine, Baltimore, MD USA; 12 Translational Medicine, The Hospital for Sick Children, Toronto, Ontario Canada; 13 Department of Medical Biophysics, University of Toronto, Toronto, Ontario Canada; 14 Mouse Imaging Centre, The Hospital for Sick Children, Toronto, Ontario Canada

**Keywords:** Doppler ultrasound, Fetal sex, Pregnancy, Pulsatility index, Sex differences

## Abstract

**Background:**

Fetal sex is known to affect pregnancy outcomes. In current clinical practice, monitoring of fetal well-being is based on Doppler ultrasound measurements of major placental and fetal vessels. The objective of this study was to investigate the effect of fetal sex on Doppler parameters throughout gestation in healthy pregnancy.

**Methods:**

A prospective study was conducted in 240 pregnant women with ultrasound examinations at a 4-weekly interval between 12 and 38 weeks of gestation. Pulsed Doppler spectra were collected for the umbilical arteries (UAs), middle cerebral artery (MCA), descending abdominal aorta (DAo), and ductus venosus (DV). Linear mixed effects models were used to determine if the pulsatility indices (PIs) of these vessels depended on gestational age and fetal sex.

**Results:**

While there were no differences in the MCA PI and DV PIV over gestation between female and male fetuses, the trajectory of the UA and DAo PIs differed by fetal sex (*p* = 0.02 and *p* = 0.01, respectively).

**Conclusions:**

Doppler ultrasound parameters were found to be dependent on fetal sex for some vessels and not for others in healthy pregnancies. Further investigations are needed to understand the physiological mechanisms for these sex differences and the relevance for disease processes in pregnancy.

## Introduction

There is a growing body of known sex differences in fetal and placental development, highlighting the importance of incorporating fetal sex as a biological variable in experimental designs and in clinical decision-making. A recent systematic review and meta-analysis concluded that fetal sex is associated with multiple maternal pregnancy complications including term preeclampsia and gestational diabetes [1]. This phenomenon is consistent with sex-specific differences in hemodynamic markers of placental and fetal maturation measured using Doppler ultrasound. Sex differences during the second half of gestation in healthy pregnancies have been reported for the umbilical artery (UA) pulsatility index (PI) [2] and the cerebroplacental and umbilicocerebral ratios [3]. Immediately prior to the onset of active labor, the middle cerebral artery (MCA) PI and umbilical venous flow were found to depend on fetal sex [4].

Ultrasound is the standard-of-care method for assessment of fetal well-being during pregnancy. Reference ranges for ultrasound-derived measures of placental and fetal health are well established [5–11]; however, these do not take into account fetal sex differences. In the present study, we investigated the effect of fetal sex on the pulsatility indices of the UA, MCA, descending abdominal aorta (DAo), and ductus venosus (DV) throughout gestation in a cohort of healthy pregnancies. Based on previous work [2–4], we hypothesized that the UA and MCA PI will depend on fetal sex. To our knowledge, no studies have looked at the effect of fetal sex on Doppler parameters throughout gestation for the DAo and DV.

## Materials and methods

A prospective longitudinal ultrasound study was conducted at Mount Sinai Hospital (Toronto, ON, Canada) and Johns Hopkins University (Baltimore, MD, USA). The Doppler spectra were acquired as part of a study that evaluated a new methodology for measuring umbilical artery hemodynamics. The latter study, which will be reported elsewhere, dictated the study sample size and inclusion criteria. We included women between the ages of 18–45 years with a healthy singleton pregnancy, a body mass index (BMI) < 45 kg/m^2^ and no significant maternal comorbidities such as type 1 diabetes or chronic hypertension. Datasets were excluded when the patient withdrew at any point during the study, the patient had preeclampsia (diagnosed according to the ACOG definition [12]) or the neonate was small for gestational age (birth weight less than the 10th centile based on neonatal sex and gestational age [13]). Patients were recruited between 10 and 17 weeks of gestation. All patients provided written informed consent to participate in the study. The study was approved by the Institutional Review Boards of The Hospital for Sick Children (Toronto, ON, Canada; REB Number 1000051548), Mount Sinai Hospital (REB Number 15-0279-A), and Johns Hopkins University (IRB Number 0082717).

Ultrasound examinations were performed on a 4-weekly interval between 12 and 38 weeks of gestation by certified research sonographers using either a Philips iU22 (Philips Healthcare, Andover, MA, USA) or GE Voluson e10 (GE Healthcare, Chicago, IL, USA) ultrasound system. Pulsed Doppler spectra were collected for the two UAs at the placental end, the distal portion of the MCA, the DAo at a position between the diaphragm and origin of the renal arteries, and the DV at the inlet portion [14]. The fetal heart rate and the PI for the UAs, MCA, and DAo were computed from the traced average Doppler waveforms as the difference between the peak systolic and end-diastolic velocities, divided by the mean velocity over the cardiac cycle. For the DV, the time average maximum velocity and forward velocities during the ventricular systole (S-wave), ventricular diastole (D-wave), and the atrial systole (a-wave) were measured and used to calculate the peak velocity index for veins (PVIV) and the pulsatility index for veins (PIV) [15]. The cerebroplacental ratio was calculated as the MCA PI/UA PI [8].

All statistical tests were performed using the R statistical software package (www.r-project.org). The two UA PI values were averaged to provide the overall mean PI. To analyze the clinical characteristics, a one-way ANOVA was used for continuous variables to evaluate the effect of fetal sex and a Pearson’s Chi-squared test was used for categorical variables. The UA PI, DAo PI, DV PVIV, and PIV data were analyzed using a linear mixed effects model with gestational age (in completed weeks), fetal heart rate, race (Asian, Black or African American, White, other), and fetal sex (female, male) as the fixed effects and a heteroscedastic random effect where inter-subject variation varied linearly with gestational age. Linear models were chosen based on previous studies of UA PI [5, 11], DAo PI [6, 9], and DV PIV [7, 10] throughout gestation. A linear mixed effects model using a second-order polynomial function was used to determine if the MCA PI and CPR depended on gestational age (in completed weeks), fetal heart rate, race, and fetal sex. A quadratic model was chosen based on previous studies [8, 11] and using a likelihood ratio test was shown to provide a better fit than a simpler linear model (*p* < 0.0001). A value of *p* < 0.05 was taken to be significant.

## Results

Two hundred and forty women consented to participate in this study. Twenty-four withdrew, 3 delivered at another site and were lost to follow-up, and 36 were excluded for adverse pregnancy outcomes (13 had preeclampsia and 23 had neonates that were small for gestational age). The 177 participants (87 females and 90 males) provided a total of 981 UA, 837 MCA, 937 DAo, and 774 DV measurements. The clinical characteristics of the participants are summarized in Table 1. The maternal demographics and pregnancy and neonatal outcomes were similar between female and male fetuses, except for the fetal weight which was significantly higher in the male fetuses (*p* < 0.001).

**Table 1:** Characteristics of the study subjects meeting the inclusion criteria. Data are mean [range] or % (n/N). ^a^*p* < 0.001 when compared to females

Characteristic	Female (*n* = 87)	Male (*n* = 90)
Maternal age at delivery (years)	34 [23–43]	34 [18–43]
Maternal pre-pregnancy body mass index (kg/m^2^)	25.2 [17.5–43.6]	25.3 [17.4–43.6]
Race (%)		
Asian	25 (22/87)	18 (16/90)
Black or African American	9 (8/87)	13 (12/90)
White	62 (54/87)	65 (58/90)
Other	4 (3/87)	4 (4/90)
Gestational age at first ultrasound visit (weeks)	16 [15-17]	16 [12-17]
Cesarean delivery (%)	36 (31/87)	41 (37/90)
Gestational age at delivery (weeks)	38 [30–41]	38 [31–40]
Birth weight (g)	3159 [1390–4390]	3422 [1410–4630]^a^

**Fig. 1:**
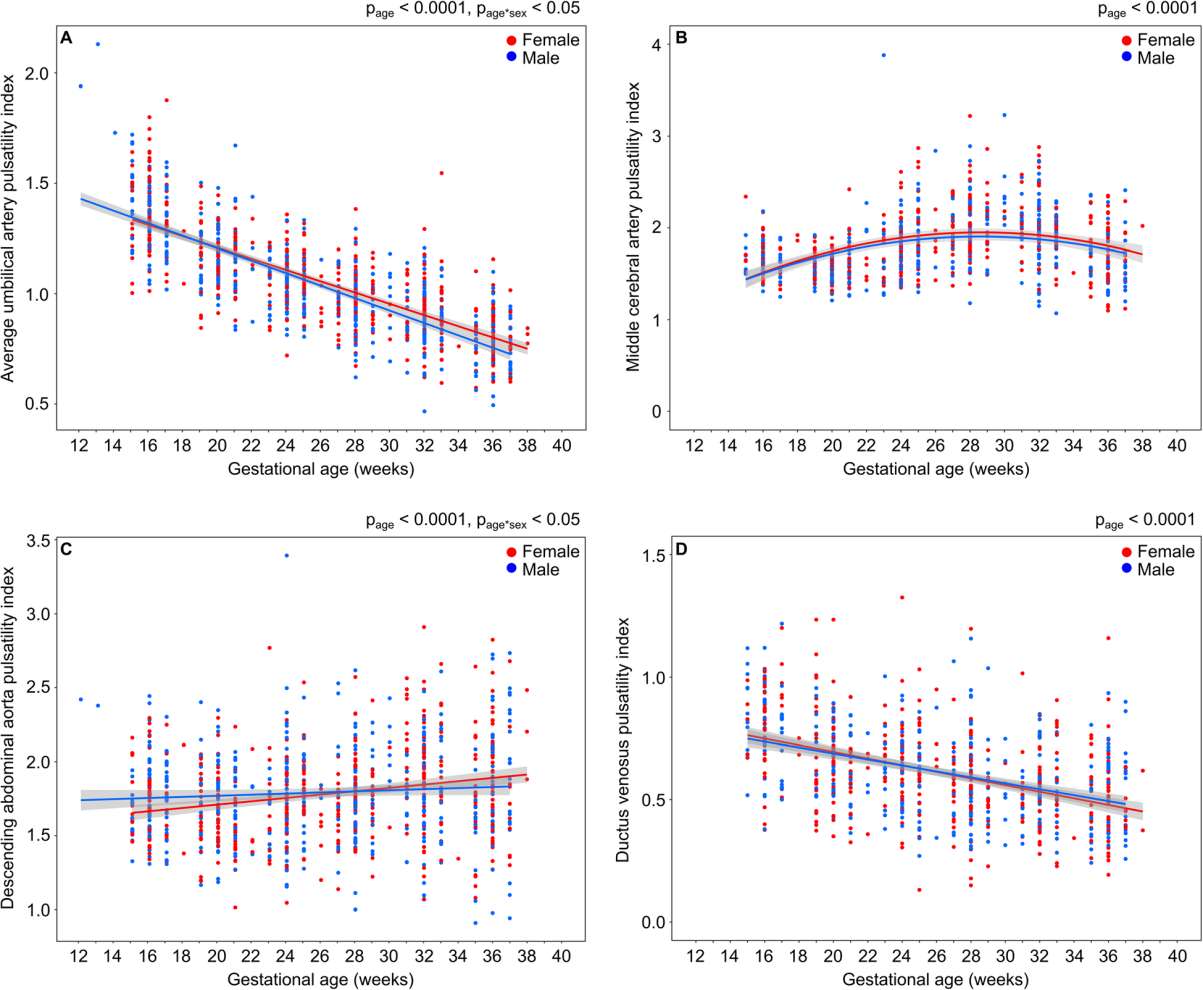
Sex differences in **a** umbilical artery pulsatility index, **b** middle cerebral artery pulsatility index, **c** descending abdominal aorta pulsatility index, and **d** ductus venosus pulsatility index for veins for female (red) and male (blue) fetuses. Main effect of gestational age as determined by a two-way ANOVA denoted as *p*_age_ and a significant age-by-sex interaction is denoted as *p*_age*sex_. The shaded gray area represents 95% confidence intervals on the fitted model

The fetal heart rate was associated with the gestational age (*p* < 0.0001) and the Doppler indices (*p* < 0.0001). While the fetal heart rate tended to be higher in female fetuses, the difference was not statistically significant (*p* = 0.06). There was no effect of race on the PIs or the trajectory of the PIs with gestation. The pulsatility indices of the UA, MCA, DAo, and DV over gestation for female and male fetuses are shown in Fig. 1. The UA PI decreased with gestational age (*p* < 0.0001) and the rate of change with gestation depended on fetal sex (*p* = 0.02) (Fig. 1a). Starting at the beginning of the third trimester, the trajectory of the UA PI for the female fetuses diverged, with significantly higher values (4% higher at term compared to males). The relationship of the MCA PI with gestational age (*p* < 0.0001) was an inverted U-shape with a turning point at 28 weeks (Fig. 1b). Similarly, the CPR varied with gestational age as an inverted U-shape (*p* < 0.0001). There was no difference in the MCA PI or CPR over gestation between females and males. The DAo PI increased with gestational age (*p* < 0.0001) and the rate of change with gestation was significantly different between females and males (*p* = 0.01) (Fig. 1c). The DV PIV decreased with gestational age (*p* < 0.0001), and there was no difference over gestation between females and males (Fig. 1d). There was also no dependence of the DV PVIV on fetal sex (not shown).

## Discussion

In the present study, we found that the pulsatility indices of the UA and DAo over gestation were dependent on fetal sex. The UA PI is routinely used as a surrogate marker of placental vascular resistance, with the goal of identifying growth-restricted fetuses that require additional surveillance [16, 17]. Sex-specific differences in the UA PI is consistent with a recent longitudinal study by Widnes et al. [2] in healthy pregnant women. While this study also found the UA PI was elevated in female fetuses compared to males, the difference was present earlier in gestation (20–33 weeks) and equalized towards term. Conversely, we found divergence of the UA PI starting at 28 weeks of gestation. This difference may be explained by the location of the measurement along the UA (placental end vs. free loop in [2]). UA PI is known to vary along the umbilical cord, with increasing resistance observed towards the placental end [18, 19]. For reliability, the ISUOG guidelines recommend using a fixed site (i.e., placental end) when collecting longitudinal measurements [20]. Another difference between the two studies is the study population. The average maternal age of our participants was significantly higher (34 vs. 30 years in [2]) and our birth weights were significantly lower (3290 vs. 3600 g in [2]), despite having a similar number of preterm births in each study (13 vs. 7 in [2]). As expected [21], the males in our study were significantly heavier at birth compared to females. While there was no difference in birth weights in Widnes et al., it has been shown that the UA PI does not depend on fetal weight [22] and therefore this difference most likely does not explain the discrepancy in the results. The difference in UA PI between females and males was small but significant (4% at term). The magnitude of this difference is comparable to previous work which reported the UA PI was 2.1–4.2% higher in females [2]. Although this difference does not suggest pathology in the female placentas, it may reflect a difference in placental vascular impedance that may explain sex-specific differences in pregnancy outcome. For example, early-onset preeclampsia (< 34 weeks’ gestation) has a higher incidence in women carrying female fetuses [23–25] and has been associated with increased resistance in the umbilical arteries [26].

Similar to the UA PI, an elevated DAo PI is associated with increased fetal risk for growth restriction [27–29]. The DAo PI showed a small but significant increase throughout gestation, consistent with previous studies [6, 9]. Moreover, the trajectory of the DAo PI over gestation differed by fetal sex with female fetuses showing a more rapid change. To our knowledge, none of the studies of DAo PI over gestation took fetal sex into account when presenting the data. In addition to providing a measurement of impedance in the placental vascular circulation, the DAo PI also represents impedance in the lower part of the fetal body. Increases in DAo PI may reflect blood flow redistribution, with blood flow being diverted away from the lower body [30]. The observation that the DAo PI changes more rapidly in females is consistent with studies in animal models that have demonstrated a greater capacity for female fetuses to redistribute blood flow [31, 32].

Another way that the fetal vasculature can respond to placental insufficiency is by a decrease in cerebral blood flow resistance, measured by the MCA PI and achieved by shunting of oxygenated blood through the ductus venosus [33, 34]. Here, the trajectory of the MCA PI over gestation was consistent with previous work that reported an inverted U-shaped curve and a turning point at 30 weeks [8]. The DV PIV and PVIV also showed the expected decrease over gestation [7, 10]. The change over gestation did not depend on fetal sex for the MCA PI, DV PIV, and DV PVIV. A study by Prior et al. reported elevated MCA PI and umbilical venous flow (which feeds into the ductus venosus) in healthy female compared to male fetuses immediately before active labor [4]. The discrepancy in findings may be related to the timing of the examination (14–38 weeks gestation vs. in labor in [4]).

Measurement of the DV waveform during the first trimester is important for screening for fetal chromosomal abnormalities [35] and major cardiac defects [36]. Previous work investigated the influence of fetal sex on DV PIV and PVIV during the first trimester. The clinical findings in healthy pregnancies were heterogeneous with DV PIV found to be elevated in females in one study [37] and not statistically different between females and males in another [38]. More recently, the Trial of Umbilical and Fetal Flow in Europe (TRUFFLE) reported assessment of the DV throughout gestation, combined with computerized cardiotocography, is critical for guiding the timing of delivery [39]. This trial motivates the use of DV Doppler waveforms throughout gestation and our results suggest considerations of fetal sex do not need to be made when using DV PIV and PVIV for clinical decision-making. The current study was restricted to a cohort of healthy pregnancies and further research will be required to establish sex differences in fetal vascular responses to placental insufficiency in a population of complicated pregnancies. Our group has recently shown that uterine artery PI in healthy pregnancies did not differ between females and males, while in pregnancies complicated by either preeclampsia, preterm birth, or fetal growth restriction, male fetuses had a significantly altered trajectory over gestation [40].

The present study had several limitations. One is that we did not collect Doppler measurements during the first trimester. Another limitation is that while we aimed to standardize the approach for the fetal ultrasound examination, the measurements were collected at two study sites and by different operators which may have introduced inter-observer variability. Finally, while the number of participants in this study provided sufficient power to test the null hypothesis, the significant differences observed between female and male fetuses in this study were small and may not be clinically relevant. However, this data is consistent with the observation of sex-specific differences in pregnancy outcomes and raises interesting questions about the biochemical and physiological mechanisms associated with the sex differences. For example, women carrying female fetuses have higher concentrations of angiotensin II than women with male fetuses [41]. Angiotensin II is known to increase blood pressure through vasoconstriction and warrants further investigation as a possible mechanism responsible for the increased vascular resistance in the DAo and UA observed in female fetuses in this study.

## Perspectives and significance

The principal finding of this work is that the trajectory of the UA and DAo PI over gestation in healthy pregnancies differed by fetal sex. Equally important, no sex-specific differences were found in MCA PI and DV PIV and PVIV over gestation. These four vessels are widely used to assess fetal well-being throughout pregnancy and these findings add to the growing evidence of sex differences in placental and fetal vascular development. Future investigations are needed to understand the underlying mechanisms for these sex differences and if these sex differences are maintained in pregnancies complicated by preeclampsia and fetal growth restriction.

## Data Availability

The datasets used and/or analyzed during the current study are available from the corresponding author on reasonable request.
